# The role of place types on social relationships and satisfaction as influenced by COVID and disabilities

**DOI:** 10.3389/fresc.2025.1624771

**Published:** 2025-07-31

**Authors:** Brent Chamberlain, Valerie Novack, Teresa Larsen, Keunhyun Park, Samuel Johnson, Jefferson Sheen, Motahareh Abrishami, Carlos Licon, Keith Christensen

**Affiliations:** ^1^ Landscape Architecture and Environmental Planning, Utah State University, Logan, UT, United States; ^2^ Department of Sociology, Utah State University, Logan, UT, United States; ^3^ Department of Forest Resources Management, The University of British Columbia, Vancouver, BC, Canada

**Keywords:** accessibility, amenities, built environment, community engagement, land use, planning, social relationships, travel behavior

## Abstract

An individual's participation in community life is important to their health, well-being, self-determination, and quality of life. Consequently, community planners and policymakers play a role in shaping and improving environments through land use planning, including the distribution of amenities. This research explores the influence of different amenities, referred to as place types, and their influence on social satisfaction during and before the COVID-19 pandemic. A nationwide online survey of 393 adults across the U.S.A. was conducted to compare participants who self-report as living with and without a disability (41% and 59%, respectively). To uncover the relationship between place types and social satisfaction, participants responded to questions about social relationships, social satisfaction, frequency of visitation to place types, and the perceived importance of place types. Results indicate that the frequency of visits and perceived importance of place types were significantly associated with social satisfaction (*r* = 0.30, *p* = 0.001). Disability status, income level, population density, and employment status significantly predicted reduced visitation frequency during the pandemic (*p* < 0.001). *Post-hoc* comparisons revealed that individuals with disabilities experience a lower level of satisfaction with social life living closer to outdoor recreation (*p* = 0.006) and healthcare facilities (*p* = 0.025) compared to other place types. The findings emphasize the need for planners to better account for accessibility and inclusion in the design and combination of community amenities.

## Introduction

An individual's participation in community life is important to their health, well-being, self-determination, and quality of life (1, 2). The design and planning of a community facilitate community participation (1, 3–7) and thus plays a role in how well individuals are connected to and supported by the community. COVID-19 (from here on referred to as COVID) upended the social fabric of our lives, partially by restricting community engagement in physical spaces—especially those that offer opportunities for community interaction (8). This reduction in engagement and face-to-face social activities has altered how individuals use amenities throughout the community. There is ample evidence demonstrating how social lives have changed, but these studies tend to highlight broad sociological phenomena (9).

While such work is foundational, few studies have empirically examined how access to specific amenities, referred to as place types (e.g., restaurants, places of work, recreation facilities, religious institutions) relates to perceived satisfaction with social life, particularly among individuals with disabilities. As we consider the role planners and policymakers have in influencing community well-being, we identify little evidence that indicates the connection between changes in social activities and satisfaction facilitated by community place types and related land use. The study contributes to this gap by focusing on a national sample to assess social satisfaction and how important place types are to social relationships amidst the COVID pandemic.

Interestingly, with the diverse knowledge and broad understanding of the linkages between social relationships and the built environment, researchers do not have a firm grasp on the relative influence that each place type, or pattern of place types, has on social relationships. Understanding how different place types affect social relationships helps planners create policies to enhance these connections. The upending nature of COVID dramatically changed how individuals used and engaged with the surrounding environment, specifically different place types within their community, which provides an ironic opportunity to evaluate how place types contribute to social relationships.

Undoubtedly, at different phases of COVID, the pandemic restricted the general population's ability to access many place types within our environment. Unfortunately, unsupportive environments can lead to isolation, social exclusion, and marginalization (5, 10–12). While COVID impacted communities broadly, one demographic that has faced more barriers to social participation is people with disabilities. People with disabilities already deal with a combination of social disadvantages which can lead to vulnerability and disproportionately adverse effects relative to the general population.

Understanding which place types hold an influence on the social relationships of people with disabilities can inform planning policy and promising practices for designing inclusive communities. To that end, the guiding research questions for this research are: (1) to what extent do people with and without disabilities perceive the importance of different place types on their social relationships, and (2) how has COVID impacted these associations for each group? In this paper, we hypothesize that COVID's impact on social relationships will be more negative among people with disabilities.

While social relationships are central to this research, they contribute toward another construct called social satisfaction. While the former typically involves understanding the presence, frequency, and structure of interpersonal ties (13), social satisfaction is typically more subjective and associated with the social network and interactions, such as the kinds of activities and local social networks (14). Social satisfaction is tied to quality of life—an important metric that planners aim to increase through policies and practices.

While social relationships are central to this research, they contribute toward another construct: social satisfaction. Whereas social relationships typically refer to the presence, frequency, and structure of interpersonal ties (13), social satisfaction is more subjective—reflecting how individuals evaluate the quality and meaning of their social interactions. It is influenced by one's social network, engagement in local activities, and broader contextual features such as neighborhood characteristics (14). Social satisfaction is tied to quality of life, a key outcome that planners aim to enhance through environmental design and policy. Indeed, research shows that built environment factors—such as access to open spaces, and mixed land uses can improve social satisfaction (15). However, the specific role of access to different place types and the frequency with which individuals visit them remains understudied, particularly for people with disabilities, whose experiences navigating social and physical environments are often shaped by barriers to access and inclusion.

This work is exploratory because of limited studies about people living with disabilities and their association with land use and related place types (where people without disabilities have been more extensively studied). Further, because no study has empirically compared a wide range of different place types and their linkages with social relationships, we anticipate the findings of this study could contribute to evidence-based decision-making and policies to facilitate land use planning and policy to create a more equitable place to live for people with disabilities. The practical implications of our findings would also be relevant post-COVID because the adjustments in travel behavior and community living might be longer-term during the “new normal”, or may have brought greater attention to long-standing disadvantages faced by people with disabilities.

## Literature review

### Connecting social activities to the built environment

The planning of a safe and healthy place with equitable (fair) access and opportunities has primarily adopted land-use allocations as a dominant implementation tool of community aspirations. The physical community environment is an important pathway to addressing social, cultural, and economic discrimination (16) and affects opportunities for community integration independent of socioeconomic and demographic variables. In particular, the built environment that provides the setting for human activity, influences an individual's community integration either positively or negatively, depending on the degree to which the environment meets the needs of the individual (1, 3, 5). The built environment mediates access to community resources, both physically and socially, necessary for participation in community life (16). A supportive environment facilitates participation in everyday activities and helps individuals build relationships that provide opportunities for self-determination and social capital (4, 6, 7). An unsupportive environment can lead to a lack of access to goods and services, isolation, social exclusion, and marginalization (5, 10–12).

Throughout the literature, there is a range of terms to qualify the meaning and value of social activities associated with various places within the built environment. Here, we use the term place types to refer to amenities that facilitate social activities (e.g., open space, restaurants, museums, etc.). Further, we introduce types of associations (e.g., social interaction, participation, and satisfaction) as a foundation for understanding how various place types afford meaning in one's social life. Social participation has been used to identify the collection of activities that individuals are involved in as a part of their daily lives (17). For instance, some place types like open spaces encourage social participation among people with disabilities (18). Social interaction relates to the repeated interaction between individuals that result in bonding and relationships. Some place types, such as restaurants, encourage interactions like social eating (19). When repeated, these activities can contribute to overall social satisfaction and attachment to certain places through place-social bonding, a phenomenon where people become attached to places that, among other things, facilitate interpersonal relationships (20). Likewise, third places (places distinct from one's home or workplace) exist within the built environment that are conducive to supporting social participation and interaction (21). These places have certain qualities to support social behavior and fulfillment including personalization, permeability, seating, and shelter (21). While social bonding, interaction, and participation provide useful ways to express connection to activities within place types, social satisfaction is used to describe the extent to which individuals feel satisfied with these expressions. Social satisfaction can be defined broadly as the contact and interactions with others (22). Satisfaction encompasses a complex space of variables, circumstances, and conditions, and has been measured through several surveys and questionnaires (14, 22, 23). For this research, we explore how place types, linked with the built environment, contribute toward an individual's social life. This enables us to quantify associations between the level of satisfaction and various place types.

Each community provides a tapestry of various amenities and activities that facilitate social connections. Community planning has had multiple approaches throughout history, with many to allocate and manage land and resources. In modern history, the US has adopted a community planning process firmly based on democratic practices, emphasizing local participation. At the core, planning practice in the US is founded under the legal grounds of promoting “health, safety, morals, or general welfare of the community” (24). Land uses are designated to promote these principles and balance equity or resolve conflicts between community and private rights. Land use planning includes a range of aspects from designating zones (e.g., residential, commercial), and conserving land and providing public resources (e.g., parks, fairgrounds). These activities facilitate the kinds of organizations, community development, and investment that create patterns of facilities and activities that give rise to social engagement. In this study, we measure social satisfaction as it relates to the different place types that citizens engage with regularly.

Our knowledge of the linkage of the built environment with social satisfaction differs dramatically between different place types. For instance, green space has been well-studied for its promotion of social interaction among urban residents (25). There is a correlation between residents' perceived amount of “greenness” in their neighborhood and their self-rating of health (26). In some communities, open space can produce a sense of belonging and bonding by providing opportunities for place-people interactions (27). While the connection between social activities with greenspace and parks may be well studied, the connection with other place types, such as grocery stores, is not as well acknowledged. Still, there are indications that grocery stores can play a role in facilitating socialization outside of the home, particularly when there aren't other opportunities (28). The level of socialization oscillates throughout the day (29).

While the amount of research drawing connections between social activities and place differs by type, we can still draw some useful context of the value that each type may have on socialization. Retail shopping, specifically malls, offers leisure and a space to interact with others because they are typically safe and comfortable (30). Workplaces can facilitate social interaction, though the design of these spaces can influence the quality of those interactions (31). Similarly, the diversity of social interactions is facilitated by perceived trust within the workplace (32). Social eating is an activity that is associated with feeling happier, more satisfied with life, and more connected to the community (19, 33, 34); we use restaurants as a place-type proxy that facilitates this activity. Community centers have also been shown to increase social participation among elderly people, improving their social satisfaction and overall health (35). More broadly, the more a person feels connected within their neighborhood (social capital), the stronger the association with social satisfaction (26). The neighborhood has shown itself to be an important social environment for the well-being of older people (36).

Everyone develops a connection to places that facilitate social satisfaction, whether those places were designed with this express purpose or not. Likewise, access to a diverse array of place types within spaces where social interaction and participation occurs could facilitate the development of third places or not, helping or hindering social interaction, participation, and overall social interaction. Inclusion in the built environment facilitates opportunities for social participation for people with disabilities (37). Individuals' access to spaces that facilitate social inclusion and participation may help reduce the social exclusion that results in lower life satisfaction for some people with disabilities. Studies have shown that for adults and youth with disabilities, social networks and activity are correlated to life satisfaction (38, 39). With the correlation between place type and social satisfaction, and the social exclusion experienced by people with disabilities due to the built environment, an analysis of how place type factors into social satisfaction can influence the design of spaces meant to facilitate inclusion.

### COVID impacts on individuals with and without disabilities

For all people, not just those with disabilities, COVID has impacted many, if not all, aspects of social and psychological health (8, 40). The necessary measures to control the spread of COVID viruses, e.g., “stay at home” orders and social distancing, have increased feelings of social isolation, depression and anxiety, and other forms of psychological distress (8, 40). These measures have also led to the increased presence of other social stressors, including financial distress (8). The combination of these factors has a direct effect on the quality of life that people experienced before the COVID pandemic and will have a lasting impact post-pandemic.

Before the COVID outbreak, people with disabilities were already experiencing concerns related to social isolation, psychological and financial distress, and mental health issues. Due to the increased risks for many people with disabilities, which includes those with chronic medical conditions, regarding COVID (41), these concerns have been exacerbated by the measures taken during the pandemic. According to the United States Center for Disease Control (CDC), it is recommended to take additional preventative steps, based on individual needs, beyond what is recommended for those who aren't considered high-risk. People who utilize direct care services have the additional stresses of navigating service providers if they become ill and must remain more vigilant in ways that those who don't use direct care must be (41).

Beyond the increased risk for many people with disabilities, other social services experience difficulties related to the pandemic. The most impacted service is medical care providers. While telehealth has become an option for many, there are still services that are only available in person and some hospitals overrun with COVID patients are unable to provide adequate services to those who need it due to regional outbreaks. The complexity of needing specific medical services but also being a high-risk individual has caused gaps in service provision, with those who have contracted COVID receiving the services.

While people with disabilities aren't necessarily experiencing more social isolation than before the pandemic, those without disabilities may have encountered a new social paradigm that has resulted in reduced social contact (42), with contact being a key component of social satisfaction (22). The issues of social isolation have created a space, potentially, for greater understanding and recognition of concerns that were not previously acknowledged by broader society. Through this study, as previously stated, we hope to gain an understanding of the impact place types have on social satisfaction for those with and without disabilities, but also how COVID may have affected each group of people differently in their experiences of social satisfaction.

## Data and methods

This exploratory approach responds to the gap in empirical research on how the built environment, particularly access and visitation frequency to place types, influences the social satisfaction of people with disabilities. The survey participants were recruited from a Qualtrics online panel. The Qualtrics platform has demonstrated a demographic representation within the U.S.A., compared with other options such as Facebook and Amazon Mechanical Turk (MTurk) (43). Previous contracts and work history with the company also helped us to secure data for this study during the pandemic.

There were no exclusion criteria for the survey (other than they were adults), but there were three demographic quotas—disabilities, age group, and gender. First, people with any type of disabilities (cognitive, mobility, sensory, etc.) should be between 40% and 50% of the sample. Second, older adults (over 65 years old) should be between 20% and 40% of the sample because they account for about a third of the national population (44). Lastly, we aimed for a gender balance between female and male—a maximum of 60 percent of either gender identity. Disability status was self-reported and participants were able to identify more than one disability type. Disability categories were identified based on the American Community Survey (2014–2018) categorizations.

We first conducted a pilot survey in late October 2020 with about 40 participants, which led us to minor changes to the survey questions, including adding a validation question at the end. The survey data included in this study were collected from November 30th to December 8th, 2020, during the U.S. winter surge, when daily new COVID-19 cases averaged around 200,000. During this period, most states had implemented varying levels of public health restrictions, including mandatory mask use in public spaces, capacity limits on businesses, and widespread remote work and learning policies. The survey was open nationally without any geographic limitations, while the respondents' locations were collected using the zip code of their primary residence.

To ensure data quality, participants were screened based on survey completion time and response consistency, resulting in a final sample size that excluded low-engagement or unverifiable cases. Specifically, participants were excluded if they completed the survey in under 6.5 min—below the first quartile of pilot completion times—or if they failed to verify their disability type in a consistency check (at the beginning and end of the survey). After screening responses, our final sample consisted of 393 respondents (161 people with disabilities and 232 people without disabilities). They live across 48 different states, and 64% of them live in a Census-designated urban area. Our target sample size was 385 respondents, calculated based on a 95% confidence level and a 5% margin of error for representing the US population of approximately 340 million people. This standard calculation for population samples determined our recruitment targets, and our final sample of 393 respondents slightly exceeded this goal.

### Survey instrument and operationalization

The final survey consisted of 60 questions and took, on average, just under 15 min to complete. The survey consists of five sections—eligibility, demographics, internet use, travel behavior, and social relationships. To align with the study's main constructs, we examined place types, interpersonal relationships, and satisfaction with social relationships among people with and without disabilities, and how COVID affected these aspects. We operationalized these themes across several sections. The survey was divided into modules reflecting these dimensions over multiple time frames (pre-, during-, and post-COVID). Questions for disability types and other demographic attributes were drawn from the American Community Survey 2014–2018 questionnaire (45), while the employment status was provided from the Critical Care Nutrition (46). The travel behavior section was analyzed in a separate study (self-citation hidden).

The social relationships section included questions that captured both the frequency of in-person interactions with family, friends, and others and satisfaction with these social relationships. To capture satisfaction with social relationships, we asked questions such as “*How satisfied are you with your current family relationships?*” repeated for friends and others outside the family and friends. Responses ranged on a Likert scale from very satisfied to very unsatisfied, and a not applicable (e.g., no relationship) option. To assess the frequency of interactions, we asked, “*How often did (would) you meet your friends in person, excluding your family members and/or people living with you?*” along with similar questions for other social groups.

To evaluate the role of place types in shaping social relationships, we asked respondents to rate the importance of various place types (e.g., house, workplace, grocery store) for the importance each has on their social relationships using a matrix question for each place type, with the question, “How important or unimportant were (or are) each of the following to your social relationships”. Respondents answered on a Likert scale ranging from extremely important to not at all important. This allowed us to examine the perceived importance of various place types for their social relationships. Each question was asked for three time periods: pre-COVID (2019), during-COVID (2020, after the outbreak), and post-COVID (a future period when COVID is no longer a significant concern in the U.S.). The survey questions were reviewed by our advisory board consisting of eight people with a variety of disabilities, and we incorporated their feedback.

### Data analysis

Several different statistical analyses were used in this study. A regression analysis was used to identify the relationship between demographic variables and the following questions: (1) “Excluding people living with you, how often did (would) you meet with any of your family members in person?” and (2) “How often did (would) you meet your friends in person, excluding your family members and/or people living with you?”. For each of these questions, we calculated the difference in scaled values between each response between their pre-COVID and during-COVID; we did not analyze post-COVID because this was not a part of this study and amid the COVID pandemic conjecture may not be a good measure of the actual future. The scales were as follows: (1) every day or almost every day, (2) 2–3 days per week, (3) once a week, (4) every other week, (5) a few times per year, (6) rarely or never. Table 1 identifies coding (including dummy coding) for the following variables used in this analysis: worker, disability, female, population density, household size, household income, and age.

**Table 1:** Mean values of variables between people with and without disabilities (*n* = 393; standard deviation in parenthesis for continuous variables).

Variables^a^	Total	No disability	With disability
Disability status dummy (1 = yes)	41.0%	-	100.0%
Most impactful disability type: sensory	-	-	31.1%
Most impactful disability type: cognitive	-	-	21.1%
Most impactful disability type: mobility	-	-	24.8%
Most impactful disability type: others	-	-	23.0%
Age (years)**	41.8 (16.9)	40.0 (15.9)	44.5 (18.0)
Age dummy (1 = 65 years or older)**	14.2%	10.3%	19.9%
Female dummy (1 = yes)*	61.3%	67.2%	52.8%
Non-Hispanic White dummy (1 = yes)**	65.9%	61.6%	72.0%
Worker status dummy (1 = full- or part-time)	50.4%	52.2%	47.8%
Educational attainment dummy (1 = bachelor's degree or higher)	32.6%	31.5%	34.2%
Household income (past 12 months)			
Less than $10,000	13.0%	13.8%	11.8%
$10,000 to $14,999	4.3%	4.7%	3.7%
$15,000 to $24,999	13.5%	11.6%	16.1%
$25,000 to $34,999	11.2%	10.3%	12.4%
$35,000 to $49,999	13.7%	12.5%	15.5%
$50,000 to $74,999	13.0%	13.8%	11.8%
$75,000 to $99,999	8.9%	9.9%	7.5%
$100,000 to $149,000	8.4%	8.2%	8.7%
$150,000 to $199,999	3.6%	2.2%	5.6%
$200,000 or more	3.6%	4.7%	1.9%
Don't know	6.9%	8.2%	5.0%
Category 1: poverty (1 = less than $25K)^b^	30.8%	30.2%	31.7%
Category 2: low (1 = between $25K and $50K)^b^	24.9%	22.8%	28.0%
Household size	2.9 (1.4)	3.0 (1.5)	2.8 (1.4)
Number of children	0.6 (0.9)	0.7 (0.9)	0.6 (0.9)
Marital status dummy (1 = married or living with a partner)	52.7%	52.6%	52.8%
Driver status dummy (1 = driver)	82.7%	81.0%	85.1%
Student status dummy (1 = student)	16.3%	16.4%	16.1%
Home ownership status dummy (1 = owner)	51.7%	51.3%	52.2%
Housing type dummy (1 = single-family housing)	63.6%	66.8%	59.0%
Population density (1,000 people/square mile; zip code)	6.3 (14.9)	5.6 (13.4)	7.3 (16.9)
Urban area dummy (1 = urban area)	64.9%	64.5%	65.6%

Note that those with disabilities may have multiple disability types.

^a^* *p* < .01, **p < .05 from an independent samples *t*-test for continuous variables and a chi-squared test for dummy variables.

^b^ Income category thresholds were selected from the U.S. 2020 poverty guideline for a four-person household ($25,701) for the “poverty” group and the U.S. Department of Housing and Urban Development's guideline of the low-income limits (80% of the area median family income) for the “low-income” group.

We then conducted a correlation analysis to identify the relationship between overall ratings of importance for our selected place types and the role COVID had on social relationships. A Pearson correlation was conducted using the mean importance rating for all place types during COVID (a five-point scale; 1-low, 5-high) and responses to the question, “How have COVID-related changes affected your social relationships?” (a five-point scale; 1-very negative, 5-very positive).

To further this analysis, we assessed the relationship between the importance rating of place type on social satisfaction and the frequency of visitation to these place types during COVID. We conducted two separate analyses, one about those living with and those living without disabilities because access can sometimes be a limiting factor for those living with disabilities (9). A Pearson correlation was used to perform these assessments across all place type ratings (five-point scale: 1-not at all important, 5-extremely important) and frequency [six-point scale: (1) every day or almost every day, (2) 2–3 days per week, (3) once a week, (4) every other week, (5) a few times per year, (6) rarely or never].

We then conducted a fixed-effects ANOVA to identify differences between individuals regarding social satisfaction and if this can be predicted by place type and disability plays. In this model, importance consisted of a five-point scale (1-not at all important, 5-extremely important), disability indicates either with or without a disability, and place type consists of all twelve distinct places. This was then followed by a fixed effects ANCOVA to understand these variables by comparing pre-COVID and during-COVID. To identify differences between place types we produced an estimate of marginal means using Fisher's Least Significant Difference (LSD) because of the implementation of the covariate with many unique place types.

To further assess the differences between pre-COVID and during-COVID, we conducted an independent samples test comparing the change between pre-COVID and during-COVID for each place type, by disability status. To accomplish this, we measured the difference in social satisfaction importance rating (pre minus during) for each place type by each individual. This result identifies the extent to which there are statistically significant changes as a result of COVID between groups.

## Results

### Demographic descriptive statistics

In our final sample of 393 respondents, 41.0% (*n* = 161) indicated at least one type of disability, which was categorized into a major disability type (sensory, cognitive, mobility, and others). Our two groups—people with and without a disability—were demographically similar except for their age, gender, and race/ethnicity (older adults, male, and non-Hispanic white people are among the respondents with disabilities). Other characteristics (employment/student status, household income, housing type, etc.) were similar between the two groups at the statistical significance level (*p* < .05). Table 1 shows mean value comparisons.

To assess the national representativeness of our sample, we compared the distribution of respondents by U.S. state to the 2020 Census population distribution. The Mean Absolute Weighted Difference was 2.63 percentage points, indicating an average weighted deviation of the sample from the Census distribution. Additionally, the Pearson correlation coefficient between the two distributions was *r* = 0.70 (*p* < 0.001), suggesting a moderately strong and statistically significant alignment between the sample and national population patterns. Several states were not sampled, with only two larger states (>10,000,000 people) not included in the sample (Georgia, and Illinois). New York and Florida were oversampled by about 13% and 6% respectively.

### Social satisfaction and visiting with friends and family

**Figure 1:**
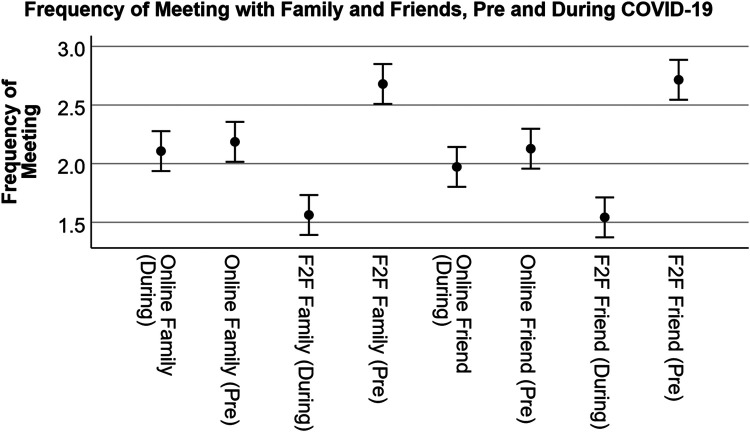
Frequency of meeting with family and friends. Frequency range = Everyday or almost everyday (5), 2–3 days per week (4), Once a week (3), Every other week (2), A few times per year (1), Rarely or never (0). Shown are average values with 95% confidence intervals.

As a central piece in this work, we surveyed overall social satisfaction. In addition to the variety of demographic data collected, we also gathered data about social relationships (e.g., the frequency and importance of visiting with family and friends). Data indicate substantial changes to the frequency of in-person visitation with family and friends, whereas online activity did not show any statistically significant difference (Figure 1). The frequency of in-person visitation changed on average from nearly weekly to about monthly.

**Figure 2:**
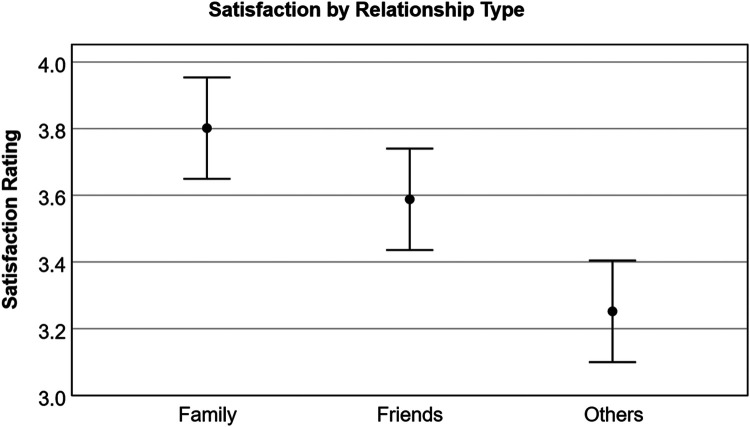
Satisfaction with relationship types during-COVID. Satisfaction Rating = very satisfied (5), somewhat satisfied (4), neither satisfied nor unsatisfied (3), somewhat unsatisfied (2) and very unsatisfied (1).

Given this difference, we were curious about the extent to which individuals associated overall impacts on social relationships as a result of COVID. The response was slightly negative with an average of 2.88 (5 = very positive impact, 1 = very negative impact). When asked about the level of satisfaction during COVID, unsurprisingly, individual responses indicated that family relationships have the highest level of satisfaction, relative to friends, while relationships with others not identified as friends have the lowest satisfaction (Figure 2; participants were given an option for not applicable if they did not have any relationships with the three types provided). Ratings were also separated by those identifying as having a disability and those not. For those identifying without a disability, social satisfaction with family, friends and other relationships was 3.68 (SD = 1.56), 3.44 (SD = 1.67), and 3.15 (SD = 1.71) respectively. Those identifying as having a disability rated family, friends, and other relationships slightly higher at 3.98 (SD = 1.30), 3.81 (SD = 1.28), and 3.40 (SD = 1.48), respectively. COVID-19 had a fairly neutral effect on these relationships; people identifying without a disability rated on average 2.84 (SD = 0.86) and those with a disability rated an average of 2.94 (SD = 0.97), where values below 3 indicate a more negative effect. These differences were not statistically significant between groups.

Given the data in Figure 1, many individuals minimized social interactions with family members and close friends. To further explore the role of various demographics, we produced a regression analysis to identify how these variables impacted the frequency of visits with family. The analysis indicates a statistically significant effect [*R*^2^ = 0.08, *F*_(7, 393)_ = 4.57, *p* < 0.001]. Table 2 provides the coefficients for each of the variables used in the regression, which indicates that household income, population density, disability, gender (female), and full- or part-time working status likely predict changes in visitation with family. The survey respondents reduced their in-person meetings with their family members further (or increased it to a lesser degree) during the pandemic when they had more household income or they were disabled, female, employed, or living in a denser area.

**Table 2:** Coefficient of demographic variables predicting change in pre-COVID and during- COVID response to the question, “excluding people living with you, how often did (would) you meet with any of your family members in person.”

Demographic variable	Unstandardized coefficients		Standardized coefficients	*t*	Sig.
	*B*	Std Err	Beta		
(Constant)	−0.287	0.359		−0.800	0.424
Age	−0.001	0.005	−0.018	−0.282	0.778
Household size	0.051	0.055	0.054	0.929	0.354
Annual household income	−0.074	0.032	−0.138	−2.323	0.021**
Population density	−0.010	0.005	−0.119	−2.249	0.025**
Disability status	−0.334	0.141	−0.123	−2.365	0.019**
Female	−0.290	0.146	−0.106	−1.983	0.048**
Worker	−0.366	0.169	−0.137	−2.170	0.031**

** *p* < .05.

Similarly, for the frequency of meeting with friends, regression analysis indicates a statistically significant effect [*R*^2^ = 0.23, *F*_(7, 393)_ = 2.84, *p* < 0.01]. Table 3 provides the coefficients for each of the variables used in the regression, which indicates that full- or part-time working status predicts changes in visitation with family (*p* < .05), while only household income and population density are the other variables that may indicate marginal influence in the outcome (*p* < 0.1). The result shows that being employed, having more household income, or living in a denser area was associated (or marginally associated for the latter two variables) with decreased social interaction with friends.

**Table 3:** Coefficient of demographic variables predicting change in pre-COVID and during-COVID response to the question, “how often did (would) you meet your friends in person, excluding your family members and/or people living with you?”

Demographic variable	Unstandardized coefficients		Standardized coefficients	*t*	Sig.
	*B*	Std Err	Beta		
(Constant)	−0.239	0.405		−0.590	0.555
Age	−0.007	0.006	−0.086	−1.309	0.191
Household size	0.026	0.062	0.025	0.420	0.675
Annual household income	−0.064	0.036	−0.107	−1.767	0.078***
Population density	−0.010	0.005	−0.102	−1.893	0.059***
Disability status	−0.117	0.159	−0.039	−0.736	0.462
Female	−0.198	0.165	−0.065	−1.199	0.231
Worker	−0.377	0.190	−0.127	−1.982	0.048**

** *p* < .05.

*** *p* < .1.

### Importance of place types on social relationships and social satisfaction

Before exploring the association between different place types and social relationships, we explored the broader relationship of the importance of relationships due to place types and how these relate to social satisfaction, during COVID. Thus, a Pearson correlation was conducted using the mean of social satisfaction across family, friends, and others, with the mean rating of importance across all place types on social relationships. There is a small-to-moderate, statistically significant positive correlation between the perceived importance of social relationships across places and overall social satisfaction (*r* = .275, *p* < .001, *n* = 393). This means that as respondents rated higher for the importance of place types on social relationships, they were also more likely to rate social satisfaction across all relationship types.

Given this result, a multiple linear regression was conducted to examine the extent to which social satisfaction was predicted by the perceived importance of social relationships across place types, disability status, and their interaction. The overall model was statistically significant, *F*_(3, 389)_ = 13.65, *p* < .001, and explained approximately 8.8% of the variance in mean social satisfaction (Adjusted *R*^2^ = .088). Perceived importance of social relationships across the place types was a significant predictor of satisfaction (*β* = .343, *p* < .001), suggesting that individuals who rated social relationships across places as more important also reported higher social satisfaction. Disability status was also a significant predictor (*β* = .118, *p* = .015), with individuals reporting a disability showing slightly lower social satisfaction overall. However, the interaction between importance and disability status was not significant.

Next, to directly assess the importance of how frequency of visits to place types and the perceived importance of social relationships was explored. A Pearson correlation assessed the mean importance rating on social relationships for all place types (for all participants) with the frequency of visits during COVID. A statistically significant moderate effect *r*_(4,323)_ = 0.30, *p* < 0.001 was found. For those reporting living with a disability, there is also a statistically significant moderate effect *r*_(1,771)_ = 0.34, *p* < 0.001, while for those without reporting a disability, there is also an effect, but the correlation was not quite as high *r*_(2,552)_ = 0.28, *p* < 0.001. This result indicates that there is an association between the frequency of visits to place types and the importance they have on social relationships. The association of access with relationships seems to be slightly higher for those living with disabilities than those without.

For the next set of analyses, the focus is on place types and social relationships. Table 4 provides the ANOVA, indicating that disability and place type are statistically significant for pre-COVID associations. However, disability shows an extremely small effect, indicating that while statistically significant, this does not explain much of the variation on place type importance (those with disability marginally indicated higher association with place types on average than those without). However, place type itself is also statistically significant, with a near moderate effect as measured by partial eta squared, indicating that there are differences in the value that each place type offers for social satisfaction.

**Table 4:** Fixed effects ANOVA for pre-COVID association of place type, visitation frequency, and disability.

Predictor	Type III sum of squares	df	Mean square	*F*	*p*	Partial *η*^2^
Corrected model	514.648	23	22.376	11.956	0.000	0.055
Intercept	35,899.080	1	35,899.080	19,180.936	0.000	0.803
Dis	8.706	1	8.706	4.651	0.031*	0.001
Place type	471.505	11	42.864	22.902	0.000*	0.051
Dis* Place type	20.129	11	1.830	0.978	0.464	0.002
Error	8,781.557	4,692	1.872			

* *p* < .05, Adjusted *R*^2^ = 0.51.

A Tukey *post-hoc* analysis shows that pre-COVID, home (either theirs or someone else's) was considered the single most important place type associated with social relationships and was statistically significant from all others (the lowest *p* = .006), whereas community service providers, educational facilities, and indoor recreation were rated lower and statistically significant from all other place types. One exception is that indoor recreation was not statistically significant from places of worship. Consider that some of these effects could be due to individuals not participating in educational institutions or having no access at all to community service providers. Most of the other place types, including online activities, were rated similarly. Figure 1 highlights these differences visually, while Figure 2 shows differences by disability status.

Table 5 provides the ANCOVA, indicating that disability, place type, and the covariate of pre-COVID (Rating Social Relationships Importance Pre) rating are statistically significant for during-COVID associations. However, disability shows an extremely small effect, similar to pre-COVID, while place type has a small effect and rating pre-COVID had a very large effect. No interaction effect was identified.

**Table 5:** Fixed effects ANCOVA for during-COVID association of place type, visitation frequency, and disability, including rating satisfaction from pre-COVID as a covariate.

Predictor	Type III sum of squares	df	Mean square	*F*	*p*	Partial *η*^2^
Corrected model	4,059.241	24	169.135	144.260	0.000	0.425
Intercept	510.611	1	510.611	435.516	0.000	0.085
Rating satisfaction importance pre	3,609.896	1	3,609.896	3,078.990	0.000*	0.396
Place type	86.412	11	7.856	6.700	0.000*	0.015
dis	5.297	1	5.297	4.518	0.035*	0.001
Place type* dis	8.799	11	0.800	0.682	0.757	0.002
Error	5,499.863	4,691	1.172			
Corrected model	4,059.241	24	169.135	144.260	0.000	0.425

* *p* < .05, Adjusted *R*^2^ = 0.422, Covariate Rating Satisfaction Importance (Pre) evaluated at = 2.80.

**Figure 3:**
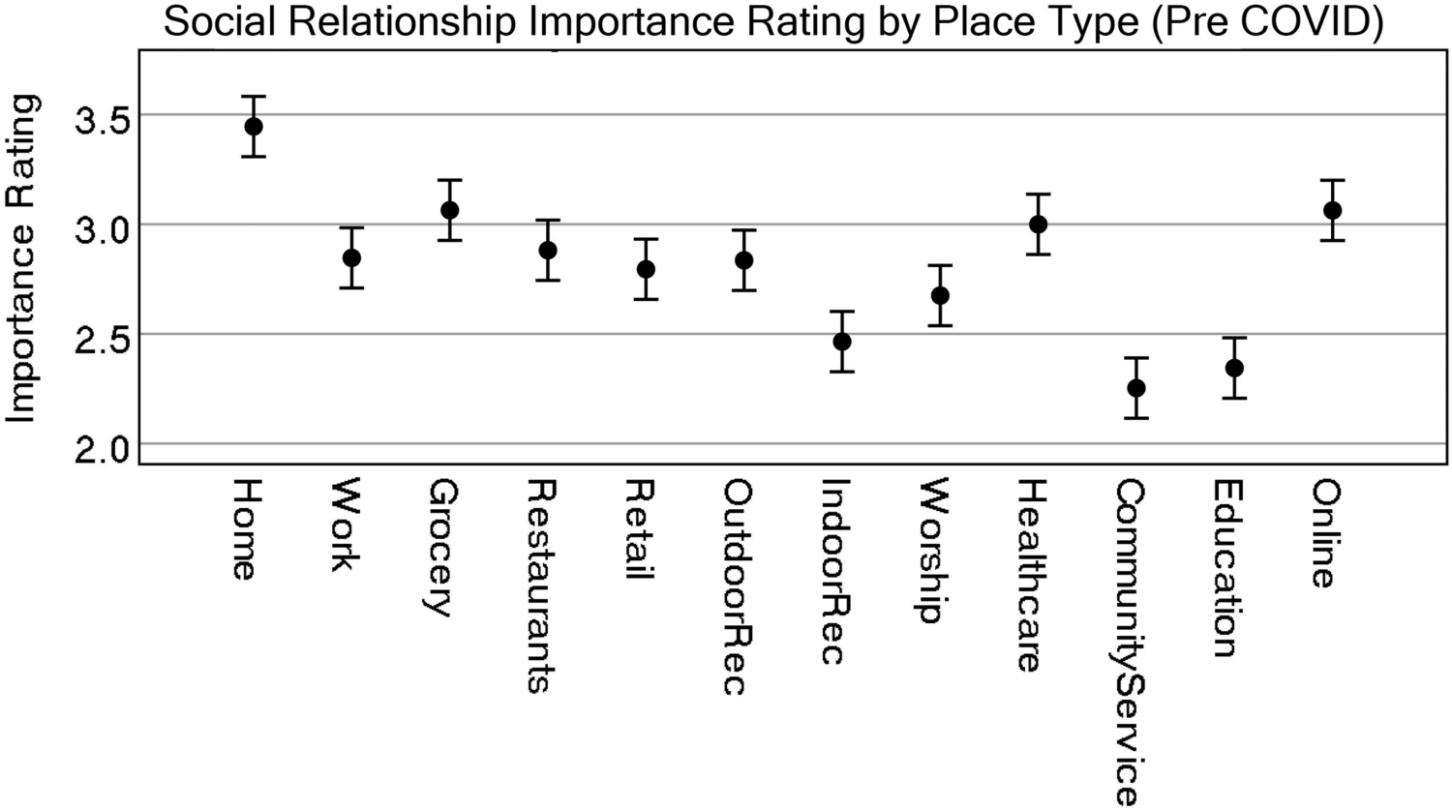
Representation of estimate of marginal means for all place types for all participants (pre-COVID). CI = 95%.

The analysis shows that online activities were considered the single most important place type associated with social relationships during the COVID pandemic and were statistically significant from all others (the lowest being grocery stores at *p* = .024 and home at *p* = .006, others *p* < .001). Importance differences between the remaining place types are a bit more nuanced than the pre-COVID model suggests. Figure 3 represents some of these differences visually, but a pairwise comparison reveals that restaurants were statistically significant to all other place types, except for retail, indoor recreation, and community service facilities. Grocery stores remained higher in importance, with no statistically significant differences between home, work, outdoor recreation, and health care facilities. Figure 5 represents the differences of the place types during COVID, after controlling for pre-COVID ratings.

**Figure 4:**
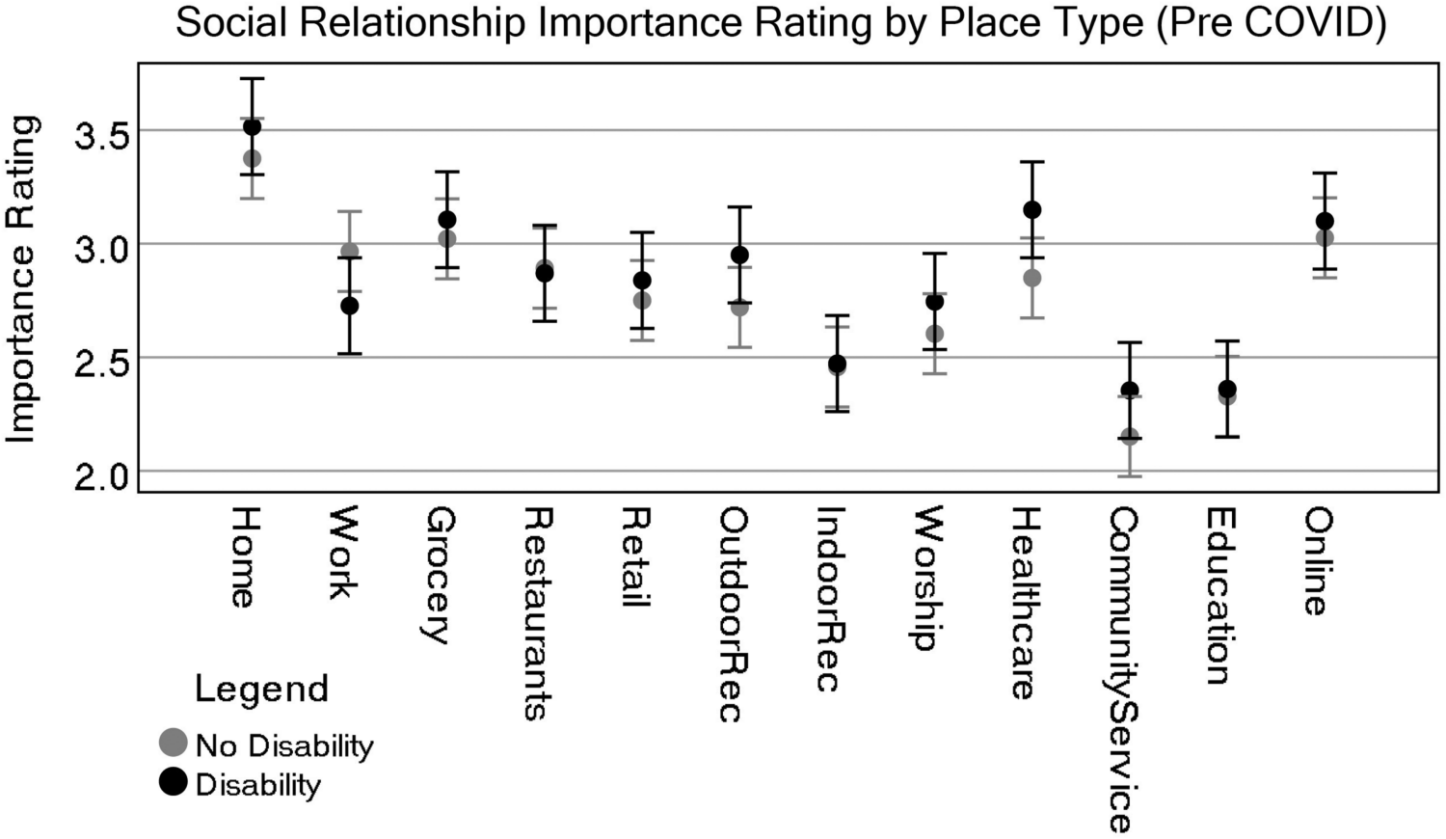
Representation of estimate of marginal means for all place types by disability status (pre-COVID). CI = 95%.

Table 4 (ANOVA) and Table 5 (ANCOVA), together with Figures 4, 6, respectively, indicated some shifts in the importance ratings of different place types and the role of disability status. To clarify the importance rating differences between each of these place types for people with and without disabilities we produced an independent *t*-test. The test was generated by first subtracting the difference from the social relationship ratings for pre-COVID and during-COVID. A Levene's test for equal variances found no statistically significant differences in variances, except Workplace (which is not statistically significant when both assuming or not assuming equal variance). Therefore, all results in Table 6 assume equal variances.

**Figure 5:**
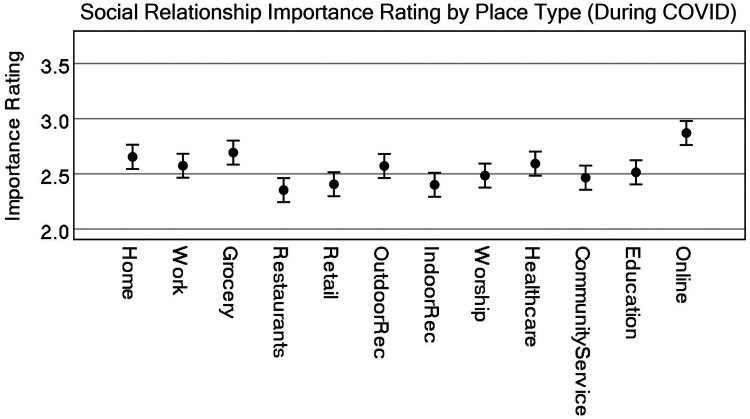
Representation of estimate of marginal means for all place types for all participants (during-COVID, after controlling for pre-COVID ratings). CI = 95%.

**Figure 6:**
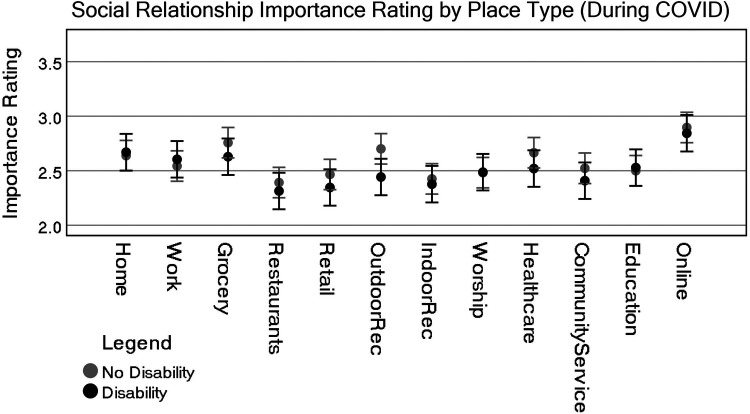
Representation of estimate of marginal means for all place types by disability status (during-COVID, after controlling for pre-COVID ratings with covariate evaluated at 2.80). CI = 95%.

**Table 6:** Welch's independent samples *T*-test showing the difference between importance of place type on social satisfaction between people with and without disabilities, where importance is the difference between pre-COVID and during-COVID ratings.

Place type	*t*	*p*	Mean difference between people with and without disabilities	Std. error difference
Outdoor Recreation	−2.768	0.006*	−0.350	0.123
Health care	−2.252	0.025*	−0.253	0.112
Come	−0.134	0.894	−0.019	0.140
Workplace	1.219	0.224	0.147	0.121
Grocery	−1.357	0.176	−0.159	0.117
Restaurants	−0.505	0.614	−0.067	0.138
Retail	−1.261	0.208	−0.151	0.120
Indoor Recreation	−0.414	0.679	−0.054	0.131
Worship	−0.406	0.685	−0.046	0.114
Community service	−1.740	0.083	−0.187	0.107
Education	0.162	0.871	0.018	0.108
Online	−0.628	0.530	−0.079	0.126

* *p* < .05, two-sided, df = 291 for all tests.

To assess whether disability status influenced changes in place importance from pre-COVID to during-COVID, we conducted independent samples *t*-tests on difference scores. Results from these tests preliminary suggest that those with disabilities indicated a statistically significant difference of importance rating for social relationships for two place types: Outdoor Recreation *t*_(291)_ = −2.768, *p* = 0.006 and Healthcare *t*_(291)_ = −2.252, *p* = 0.025. Additionally, Community Service facilities demonstrate evidence that this could play a role in the importance of social relationships, though only significant at *p* < 0.1; *t*_(291)_ = −1.740, *p* = 0.083. All other tests for place type by disability showed no statistically significant differences. The results indicate that individuals with disabilities reported significantly lower ratings of importance for place types during the pandemic, with the most pronounced declines for locations requiring physical access, such as outdoor recreation and healthcare facilities.

## Discussion

This study represents one of the first attempts to quantify the role that place types have on social relationships. In this exploratory work we sought to answer two research questions: (1) to what extent does each place type impact social relationships for people with and without disabilities, and (2) how has COVID impacted these associations for each group? We hypothesized differences between disability status across place types. Our approach to this research focused on a generalized form of place types, to uncover if any differences exist across categories. In the sections below, we discuss and reflect on specific findings.

### Reflection on place types, social relationships, and social satisfaction

Our findings suggest place types garner different associations with social relationships both pre-COVID and during-COVID pandemic. Some of the place types indicated a low association with social relationships, such as indoor recreation, community service providers, and education facilities. The low association of these may be due more to the functional choices (e.g., do not use a gym or educational activity), rather than social choice. Yet, there are indications that some place types were valued more for their association with social relationships than others. Certainly home (pre-COVID) and the use of internet (during-COVID) show important associations. Also, there were other obvious differences in place types like grocery stores, restaurants, and retail. Apparently, with the restrictions in place on operating retail and restaurants, this policy directly reduces the association of these place types with social satisfaction.

Additional and future exploration of the similarities and differences in place types is warranted. The survey results confirmed associations between social relationships and place types, but details about how place types were being used and for what purpose were not asked of participants. It would be useful to prod these questions because they may have implications for behavioral patterns or limits that differ between people with and without disabilities. These differences could be associated with attributes like accessibility (for people with disabilities), even though it may not be as high of quality. For instance, large brand-name stores and restaurants may be physically more accessible to people with mobility disabilities but may lack other desirable qualities of smaller local restaurants and shops that are unable to provide adequate space or compete for the most accessible location. Further, social routines, habits, and cognitive limits can limit and inculcate the choice of places we visit (47). Identifying why individuals choose certain place types can enable researchers to determine if there are specific designs, atmospheric, location (transport access), or other features that influence the quality of place types.

As outdoor recreation is strongly associated with an individual's physical, psychological, and social health (48–51) one of the more important findings from this research is the role that outdoor recreation had with its association of social relationships. People with disabilities indicated a decline of importance in outdoor recreation facilities from pre-COVID to during-COVID on their social relationships. Similarly, a Scottish transportation study on outdoor exercise during phases of the COVID lockdown found participants who identified as having a “health problem or disability” or being aged 65 + were significantly less likely to have participated in outdoor recreation than other groups of participants. In their study, access to a personal vehicle accounted for at least some of the gap, as those with access to vehicles were more likely to participate in outdoor exercise than those without personal vehicles (52) Additional studies have linked personal vehicle access and social satisfaction (23) and noted the inequitable distribution of public parks in walking distance of under-resourced neighborhoods (9). The devaluing attitudes of people without disabilities towards people with disabilities, often called ableism, also have been found to affect people with disabilities' feelings towards travel (53–55). For places and events that do not have a hybrid option, timely and inclusive public transit may help address gaps where older people and others with disabilities do not participate due to issues of access, safety, and lack of inclusive travel options.

One of the other place types that saw a statistically significant drop in importance for social relationships, was health care. The difference in importance rating pre-COVID and during-COVID was different only for those living with disabilities. This study did not explore the reasons why this effect happened, but the literature suggests some probable reasons. For instance, many people with disabilities are considered high-risk and are quite conscious of their social exposure potential (56). Thus, more precautions may have been taken to visit healthcare facilities because of existing conditions and the perception of providers being places of higher risk than pre-COVID (57). Further, increased anxiety about health and isolation (58), relative to those without disabilities, could have also played a role in reducing the importance of these place types. Finally, another potential cause in the association could be due to healthcare rationing policies at providers (59). Unfortunately, people with disabilities have a greater regular reliance on health care providers, and building trust and relationships can be important for care—thus the reduction in importance of social relationships of these place types represented another disproportionate effect on people with disabilities. Changes to healthcare systems, including the broadening of telehealth practice, may continue to provide additional healthcare access to people with disabilities increasing social relationships in the future (even if they are virtual). Future research on relationships and healthcare as a place type could provide beneficial information in healthcare facility planning and development.

More broadly, unsupportive environments can lead to isolation, social exclusion, and marginalization (5, 10–12). While certain retail locations aid in addressing loneliness and its effects (60, 61), restaurants and commercial spaces can be places that individuals frequent as a result of social isolation, with old age being a frequent population studied for this phenomenon. Chronic illness, disability, and old age, among other factors, contribute to the loss of relationships that may increase the use/benefit of these place types in the future. Older adults with disabilities have been found to associate social participation with life satisfaction (39). One of our findings supports these insights for people living with disabilities. We found that social relationships were moderately correlated with the frequency of visits to place types, whereas this effect was still significant for people without disabilities, but less so. Further research into additional place types and their importance with social relationships and frequency of visits could lead to a better understanding of the successful planning of zoning and land use and the proportion of certain place types in development.

### Social satisfaction, social relationships, and the built environment

While place type association and social relationships seem like a relevant link, social satisfaction also pertains to several relationship factors that may not be directly related to the built environment. For instance, visiting family and friends (Tables 2, 3) offers a few potential interpretations of the linkages between these critical relationships (62, 63). Many people with disabilities utilize professional caregiving services as part of their daily lives. As the pandemic progressed, there were growing issues accessing care provider services. Many providers before COVID were experiencing worker shortages and difficulty retaining their workers. Once those services were complicated through exposure, illness, and deaths, many people lost their caregivers. Family may have stepped into filling some of the caregiving roles previously managed through a care provider. This means that during the pandemic, family members may have visited more frequently. This particular circumstance, however, cannot be confirmed through this study, merely speculated. The importance, reliance, and possible dependence on family for social satisfaction could explain the small change in social satisfaction pre-COVID and during-COVID (even though these were statistically significant). Visiting with friends, however, did not demonstrate a change pre-COVID and during-COVID based on disability status. As friendships and relationships with family are typically different in their overall role expectations, regardless of disability status, those with disabilities may not be relying on their friends for the same kind of support during the pandemic as they are relying on their families. Family members may be providing more in-person support than friends.

Our analysis indicates household income as a significant factor in decreasing social interaction with family and friends, the suggested behavior in response to the coronavirus. Income also plays a factor in the use of informal caregiving. In the U.S. the number of adults acting as nonpaid caregivers hit above 20% in 2020, with lower-income households having higher use of non-related unpaid caregiving (64). It is possible persons with lower income and disabilities were unable to decrease social interaction with friends and family members who they rely on for certain tasks related to daily living, as well as differences in remote workability.

### Future work

This study used a broad definition of place types to uncover if individuals perceived differences between these places and their social relationships. While we observed some distinct differences, we recognize the need to further investigate the role different qualities of place types may have on social relationships and, as well, social satisfaction. For instance, different kinds of restaurants, retail, or community service providers can vary significantly, potentially leading to different associative meanings for social relationships, which can influence their overall social satisfaction. Further, the degree to which place types are accessible or universally designed may impact satisfaction. The accessibility of transportation, such as with public transit and streets, has been linked with social satisfaction (23), and the likelihood that the accessibility of place types is also linked with social relationships should be examined in future research. Another important future development is to explore how the diversity and configuration of these place types may also play a role. To this end, it would be imperative to map the extent of places that individuals visit, and importantly what kinds of places are accessible within a meaningful distance of their primary residence.

Research that is based on self-reported associations provides a direct window into individual sentiment, but this can make it difficult to interpret the cause of these sentiments. Likewise, requesting that individuals recall and foreshadow sentiments and activities can be difficult. In future work, and now with ongoing COVID, we anticipate developing longitudinal studies to improve the quality of data collected. Nevertheless, subjective responses have been used in several studies (65–68), and we believe that this is a relevant and direct measure to evaluate social satisfaction. When combined with future augmentations of the quality, configuration, and accessibility of place types, we hope to further deepen our understanding of how the built environment, and the policies that inform its development, influence social satisfaction for people living with and without disabilities.

## Conclusion

This exploratory research aimed to identify potential associations between social relationships and the impact of different place types on these relationships, as well as overall social satisfaction, which is a measure of quality of life. Place types are a natural outcome of different zoning and land-use planning policies, thus identifying linkages to social relationships with the built environment can aid in supporting policies that improve social relationships and social satisfaction. Further, this research aimed to explore the role that COVID had on the associations with place types and social relationships, but more importantly to identify differences between people living with and without disabilities. Our findings indicate that place types have a role in influencing social relationships and that these roles may differ slightly between those living with and without disabilities—particularly for important places like health care and outdoor recreation places. We hope the findings of this study can contribute to evidence-based decision-making and policies to facilitate land use planning and policy to create a more equitable place to live for people with disabilities. Continuing research on what characteristics of place types affect social relationships or facilitate social interaction can inform work being done in place value, place human-centered design of public spaces and neighborhoods (69). Practical implications of our findings would also be relevant post-COVID because the adjustments in travel behavior and community living might be longer-term or have brought greater attention to long-standing disadvantages faced by individuals with disabilities.

## Data Availability

The raw data supporting the conclusions of this article will be made available by the authors, without undue reservation.
